# Painless lump over the forehead which turned painful: an unusual presentation of hepatocellular carcinoma

**DOI:** 10.1259/bjrcr.20150033

**Published:** 2015-05-18

**Authors:** S P Susheela, S Revannasiddaiah, A S Basavalingaiah, I Madabhavi

**Affiliations:** ^1^Department of Radiation Oncology, HealthCare Global, -Bangalore Institute of Oncology, Bengaluru, India; ^2^Department of Radiation Oncology, Government Medical College-Haldwani, Nainital, India; ^3^Department of Medical & Pediatric Oncology, Gujarat Cancer Research Institute, Ahmedabad, India; 0000-0001-6985-9874

## Abstract

Hepatocellular carcinoma (HCC) is one of the most common malignancies and is known to most often present with symptomatology pertaining to local hepatic disease. Although HCC is known to metastasize to lungs, abdominal lymph nodes, adrenal glands and the vertebral column, it is rather rare to come across patients with skull metastasis. The manifestation of a solitary frontal bone metastasis leading to a painless lump over the forehead as the initial presenting feature of HCC is highly unusual. This case report pertains to a 40-year-old male patient who had initially observed a painless lump over his forehead that was gradually increasing in size over a span of 3 months. He sought medical attention when, after several months, the “painless lump” suddenly became painful. Investigations revealed the involvement of both the outer and the inner table of the frontal bone, and a biopsy revealed the histopathology to be that of HCC. On further investigation, he was found to have systemic disseminated disease involving both the left and right lungs and vertebrae and treatment was initiated with sorafenib. Despite an initial partial response, the patient subsequently succumbed to hepatic failure. This case report illustrates the fact that HCC can silently progress, and even lead to dissemination and distant metastases before becoming clinically evident.

Hepatocellular carcinoma (HCC) is one of the most common malignancies in the world, with an enhanced prevalence in Asia and Africa.^1^ Its association with chronic viral hepatitis (hepatitis B and C) is the reason behind the higher prevalence of HCC in Asia and Africa than in the Western world. Although a majority of patients with HCC present with features of localized hepatic disease, metastatic disease is not uncommon. Although HCC is known to metastasize to lungs and regional lymph nodes, skeletal metastases are relatively uncommon. When skeletal metastases from HCC occur, the bones affected commonly are those of the vertebral column, pelvis and the ribs. Skull metastases are rather rare.^2–4^

It has been recently claimed that bone metastasis from HCC is now showing an increasing incidence in comparison to previously. HCC was often associated with very short survival in the past, as patients would succumb to primary liver cancer even before metastatic disease could occur. However, with the advent of better surgical techniques, radiofrequency ablation, stereotactic radiotherapy, intra-arterial chemotherapy and sorafenib, many patients with HCC now survive longer, and thus distant metastases, including skeletal metastasis, are being reported more commonly of late.^5–10^

This current report describes the unusual case of a patient who presented with manifestations of skull (frontal bone) metastasis as the initial presenting feature of HCC. The current case illustrates that it is possible for a patient to develop silent systemic dissemination before the patient becomes symptomatic owing to HCC. Also, the manner of presentation is rare, with the patient experiencing a seemingly innocuous symptom of a painless lump over the forehead, which turned painful after 3 months, only to be diagnosed as metastatic HCC.

## Case Presentation

**Figure 1.**
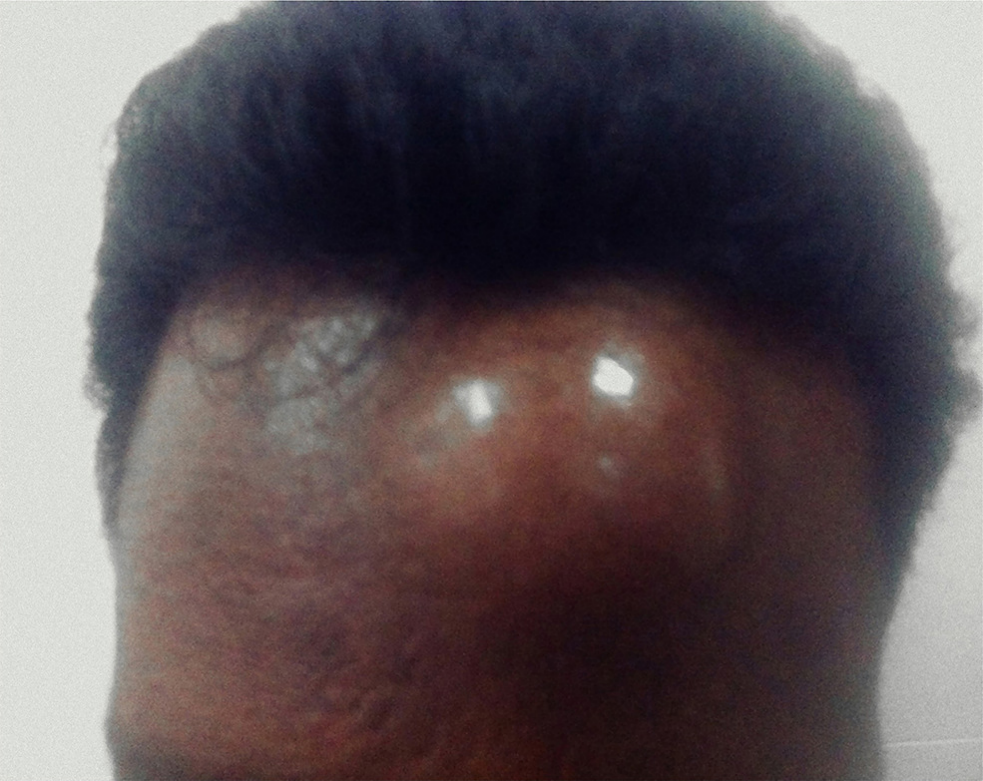
Clinical photograph of the patient at presentation, demonstrating the “lump” on the forehead.

**Figure 2.**
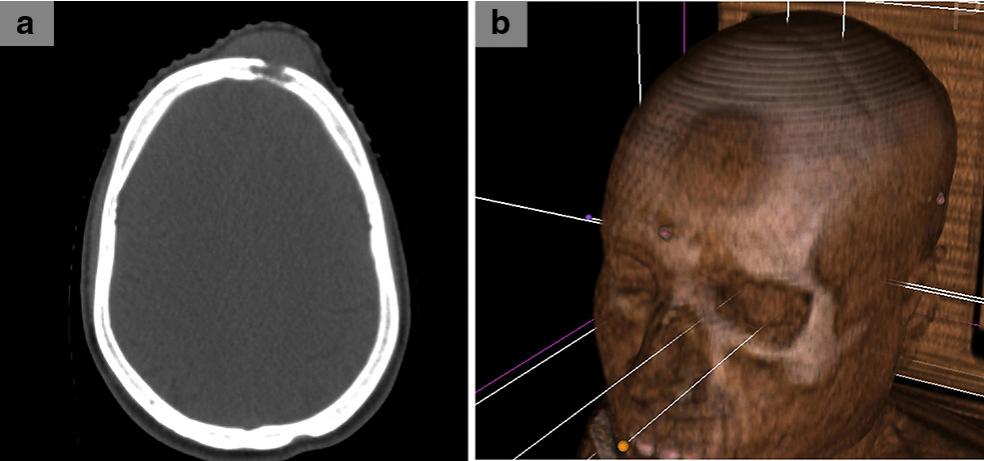
Axial CT slice (a) demonstrating the frontal bone metastasis involving both the outer and inner tables of the bone; three-dimensional reconstruction (b) of the lesion demonstrating the location within the frontal bone.

**Figure 3.**
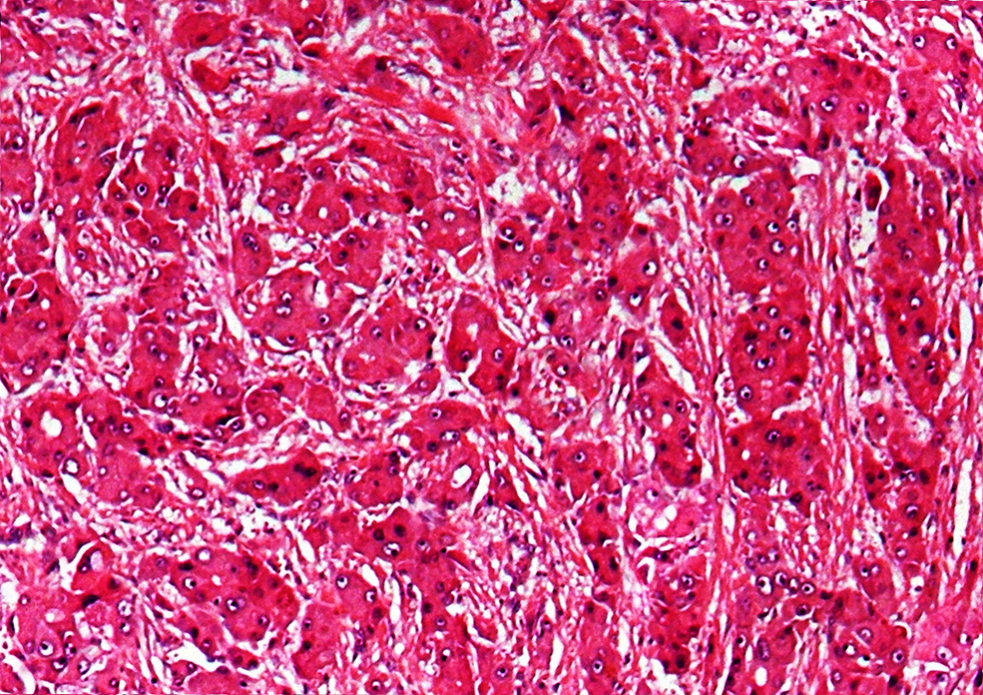
Haematoxylin and eosin histopathology revealing pleomorphic tumour cells having predominantly eosinophilic cytoplasm, prominent nucleoli and cells being arranged in both trabecular and solid patterns.

A 40-year-old male patient presented to us with a 3-month history of having noticed a painless lump over his forehead. He reportedly ignored the lump, initially assuming it to be a pimple, but became concerned when it continued to grow gradually. He stated that it turned painful, and that was when he came in seeking medical attention. On examination, the lump (Figure 1) over the frontal region was hard, fixed and tender on palpation. CT scan demonstrated that the lesion involved both the outer and the inner tables of the frontal bone (Figure 2). Upon core needle biopsy, the lesion on haematoxylin and eosin staining (Figure 3) demonstrated pleomorphic tumour cells having predominantly eosinophilic cytoplasm, prominent nucleoli and cells arranged in both trabecular and solid patterns.

**Figure 4.**
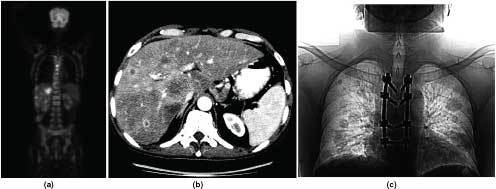
(a) Coronal positron emission tomography scan slice revealing highly avid lesion in the liver, thoracic vertebrae as well as less avid lesions in the lungs; (b) multiple lesions in the liver on CT scan; (c) post-vertebral fixation image revealing multiple cannonball metastases in both lungs.

Concurrently, a whole body 18-fludeoxyglucose positron emission tomography (^18^F-FDG-PET) scan was performed to look for potential sites of primary tumour. This revealed a very highly avid lesion (standardized uptake value of 28) in the right lobe of the liver, along with multiple other avid foci involving the liver, lungs and the dorsal vertebrae. The lesions in the liver, lungs and dorsal vertebrae were also visualized on digital reconstruction of CT images (Figure 4).

Thus, further investigations were focused upon confirmation of HCC as the primary tumour. The patient’s α-fetoprotein (AFP) level was found to be 6889 ng ml^−1^. Immunohistochemistry showed positivity for HepPar1 and CD10. Negative staining was noted for TTF-1, CEA, ALK-1, LCA, S-100, vimentin and chromogranin. The patient was seropositive for HBsAg and negative for HCV. The patient reportedly was unaware of his prior HBsAg status. His liver functions tests were within normal limits at presentation. Serum CEA, CA 19-9, coagulation tests, liver function tests and other routine investigations were normal at presentation. The patient was a driver by occupation, and as a non-drinker and non-smoker. There was no history suggestive of aflatoxin exposure. There was no history of blood transfusions.

Given the detection of multiple lung and vertebral metastases, radical curative surgery and chemotherapy were not considered. The patient's Karnofsky Performance Status (KPS) at diagnosis was 70.^11^ After fixation of vertebral metastasis (Figure 4c), his involved vertebrae were irradiated with a dose of 20 Gy in five fractions with 6-MV beam by a single direct field, calculated using the skin–source distance of 100 cm with a depth of 4 cm. He was then treated with sorafenib (initiated at a dose of 400 mg, twice a day), which was tolerated well without any remarkable toxicity. He was also treated with oral ibandronate (150 mg, once a month) and oral analgesics (non-steroidal anti-inflammatory drugs, tramadol and gabapentin).

There was a clinically visible regression of his forehead lesion (it must be emphasized here that the forehead lesion was not irradiated, and the option of irradiation was reserved for use in case of non-response with sorafenib). Furthermore, there were minimal regressions/stabilizations in the sizes of the thoracic metastases, secondaries elsewhere as well as in the primary. The use of sorafenib had also led to an improvement in his performance status for a span of 6 months (from a pre-treatment KPS value of 70 to approximately 80–90 during the first 6 months while on sorafenib). However, despite the initial response after 6 months of initiation of sorafenib, the patient had progression of his lesions as well as the appearance of new lesions. Although sorafenib was continued, the patient ultimately succumbed to hepatic failure a year after diagnosis.

## Discussion

HCC is mostly a result of chronic damage repair, which is characteristic of conditions such as viral hepatitis (owing to hepatitis B and C), alcoholic cirrhosis, autoimmune hepatitis, intake of aflatoxin, primary biliary cirrhosis, sclerosing cholangitis and many others. In Asia and Africa, a significant proportion of the burden of HCC is attributable to chronic hepatitis owing to hepatitis B and C viruses. It is often reported that HCC with underlying viral aetiology often presents at a younger age than in non-viral-associated HCC.^2,12^

In the past, the majority of HCC patients would succumb quickly to hepatic failure owing to progressive malignancy within the liver. However, the advent of surgical techniques, including liver transplantation, the advent of intra-arterial chemotherapy, and additional techniques including radiofrequency ablation, stereotactic radiotherapy etc. have led to an increase in the average life expectancy of HCC patients.

The incidence of bone metastasis in HCC is quite rare, and is estimated to range from 2% to 16%.^4,13^ The most common sites of skeletal metastases include the vertebrae, ribs, sternum and the long bones. However, it is notable that metastasis to the skull is very low, with an incidence of 0.5–1.6%.^14–16^ Although rare, there has been published literature that has attempted to explain the route of HCC metastasis to the skull.^14–16^ It is regarded as spreading via the “osseous route”, that is, the metastatic vertebral involvement could lead to the spread of metastases via the Batson’s venous plexus to the skull. Once the skull is reached, the cells disseminate within the diploic venous channels and then proceed to erode both the inner and outer tables of the cranial bones.^14^ This theory is indeed supported by our case, wherein vertebral metastases were found, as well as skull metastases with involvement of both the inner and outer tables being seen.

In a recent literature review published by Guo et al,^16^ the issue of skull metastasis from HCC has been addressed. They were able to find a total of 59 patients with skull metastases from HCC. They noted an increasing trend towards the reporting of skull metastases from HCC from the year 1990 onwards. The authors also noted that, of these 59 patients, 24 cases presented with skull metastasis as the first symptom of HCC. An alarming fact was that 71% of these 24 cases were initially misdiagnosed, mostly attributable to the lack of clinical suspicion as to the possibility of HCC being the reason behind skull metastasis.^16^

The patient being described in our case indeed could be diagnosed accurately as having metastatic HCC because of the availability of modern diagnostic assistance. However, it is notable that the patient himself had for the initial 3 months ignored the lump as trivial as it was painless. Only when it became painful, was medical attention sought by him. The patient could not be considered for resection of metastases as there was presence of systemic disseminated disease at diagnosis. Sorafenib, although known to be of benefit in metastatic HCC, was not of much benefit in our patient.

In conclusion, this case illustrates the rare presentation of a patient with a forehead lump owing to frontal bone metastasis as the initial presenting feature of disseminated HCC. The adage that “cancer is a great masquerader” is indeed true. The presentation of malignancies is often vague and obscure, and given the lack of alarming symptoms in the earlier stages may cause patients to present in advanced, often incurable stages. Given the very high incidence of viral hepatitis-induced HCC in Asia and Africa, it may be prudent to include routine screening of populations for seropositivity for HBsAg and HCV. Also, it may be worthwhile providing frequent screening with imaging and serum AFP measurements for patients known to be seropositive for viral hepatitis so as to detect HCC in an early, curable stage.

## Learning Points

1. Cancer is a proven masquerader and often presents in a way resembling more common benign diseases.
2. Skull metastases from HCC are rare, but may occur from vertebral metastases via the Batson’s venous plexus.
3. Although highly unlikely to result in long-term survival, the use of sorafenib can provide reasonable improvements in progression-free survival.
4. Given the very high incidence of viral hepatitis-induced HCC in Asia and Africa, it may be prudent to include routine screening of populations for seropositivity for HBsAg and HCV.
5. It may be worthwhile to provide frequent screening with imaging and serum AFP measurements for patients known to be seropositive for viral hepatitis so as to detect HCC in an early, curable stage.
